# *Datura Metel* L. Ameliorates Imiquimod-Induced Psoriasis-Like Dermatitis and Inhibits Inflammatory Cytokines Production through TLR7/8–MyD88–NF-κB–NLRP3 Inflammasome Pathway

**DOI:** 10.3390/molecules24112157

**Published:** 2019-06-07

**Authors:** Bing-You Yang, Yan-Gang Cheng, Yan Liu, Yuan Liu, Jin-Yan Tan, Wei Guan, Shuang Guo, Hai-Xue Kuang

**Affiliations:** Key Laboratory of Chinese Materia Medica, Ministry of Education of Heilongjiang University of Chinese Medicine, Harbin 150040, China; yangbingyou@hljucm.net (B.-Y.Y.); chengyg1992@163.com (Y.-G.C.); lifeliuyan@hljucm.net (Y.L.); flyliuyuan@163.com (Y.L.); 15636833827@163.com (J.-Y.T.); myguanwei1234@yeah.net (W.G.); 18231176108@163.com (S.G.)

**Keywords:** psoriasis, *Datura metel* L., imiquimod, inflammatory cytokines, toll-like receptor 7/8

## Abstract

Background: Psoriasis is a chronic, immune-mediated inflammatory skin disease, and the inflammatory response plays an important role in its development and progression. *Datura metel* L. is a traditional Chinese medicine that exhibited a significant therapeutic effect on psoriasis in our previous study due to its remarkable anti-inflammatory effect. Meanwhile, the mechanism underlying its effects on psoriasis is still unclear. Methods: An imiquimod-induced psoriasis-like dermatitis mouse model was constructed to evaluate the protective effect of the effective part of *Datura metel* L. (EPD), which was verified by evaluations of the Psoriasis Area and Severity Index (PASI) score. Hematoxylin and eosin (H&E) staining, immunohistochemical examination, enzyme-linked immunosorbent assay (ELISA), and Western blot were used to measure the inflammatory cytokines and the protein expression associated with the Toll-like receptor 7– myeloid differentiation primary response gene 88–nuclear Factor-κB–nucleotide-binding oligomerization domain (Nod)-like receptor family pyrin domain-containing 3 (TLR7/8–MyD88–NF-κB–NLRP3) inflammasome pathway. Results: EPD significantly decreased the PASI, reduced epidermal thickness, and decreased the proliferation and differentiation of epidermal cells in psoriasis-like dermatitis C57BL/6 mice induced by imiquimod (IMQ). Furthermore, EPD reduced the infiltration of CD3+ cells to psoriatic lesions, as well as ameliorated the elevations of intercellular adhesion molecule 1 (ICAM-1) and inhibited the production of imiquimod-induced inflammatory cytokines, including IL-1β, IL-2, IL-6, IL-10, IL-12, IL-17, IL-22, IL-23, tumor necrosis factor-α (TNF-α), monocyte chemotactic protein 1 (MCP-1), and interferon-γ (IFN-γ). Besides, EPD decreased the imiquimod-induced expression levels of TLR7, TLR8, TRAF6, MyD88, p-IKKα, p-IKBα, p-NF-κB, NLRP3, apoptosis-associated speck-like protein contained a caspase recruitment domain (ASC), cysteinyl aspartate specific proteinase 1 (caspase-1), and IL-1β. Conclusion: This study demonstrated that EPD exhibited a protective effect on an imiquimod-induced psoriasis mice model by inhibiting the inflammatory response, which might be ascribed to the inhibition of the TLR7/8–MyD88–NF-κb–NLRP3 inflammasome pathway.

## 1. Introduction

Psoriasis, a chronic inflammatory skin condition, is commonly considered to be induced by multiple environmental and genetic factors. It is characterized by scaly reddish plaques because of the hyperproliferation and aberrant differentiation of keratinocytes [1]. Psoriasis has been estimated to globally affect approximately 2% to 4% of the population [2]. Meanwhile, besides affliction by a skin disorder, psoriatic patients might have a higher incidence of cardiovascular disease, diabetes, arthritis, depression, and even cancer [3,4,5,6,7]. These conditions had a significant impact on the physical and psychological health of psoriatic patients, and have also caused tremendous socioeconomic and psychological burden [8,9].

The exact pathogenesis of psoriasis is unclear, but it is commonly believed that the inflammatory reaction and abnormal activation of immune cells functions played the key roles in the onset and development of psoriasis. Excessive inflammatory factors activated multiple intracellular signaling pathways and stimulated transcription factors; thus, the cytokines released by immune cells dramatically increased, and the epidermal symptoms proliferated, finally leading to the aggravation of psoriasis. Toll-like receptors (TLRs) are pattern recognition receptors that play important roles in the development of psoriasis. Generally, the main function of TLRs was to mediate the inflammatory response [10,11]. TLR signaling involved the recruitment of adaptor proteins; TLR7/8 were the main TLRs which induced the recruitment of myeloid differentiation primary response gene 88 (MyD88); then, the MyD88-dependent pathway activated nuclear Factor-κB (NF-κB), and this caused various inflammatory cytokines to extensively express, as well as provided the positive feedback effect on the TLR7/8-MyD88-NF-κB signaling, thus resulting in the persistent inflammation [2,12]. Additionally, as one of the downstream factors of TLRs, the nucleotide-binding oligomerization domain (Nod)-like receptor family pyrin domain-containing 3 (NLRP3) inflammasome is a cytoplasmic macromolecular complex and consists of NLRP3, cysteinyl aspartate specific proteinase 1 (caspase-1), and apoptosis-associated speck-like protein contained a caspase recruitment domain (ASC). The activated NLRP3 inflammasome could result in the activation of ASC, caspase-1, and the release of mature IL-1β, and eventually promoted the development of inflammation [13]. Accordingly, the pathogenesis and progress of psoriasis were closely related to TLR7/8-MyD88-NF-κB and NLRP3 pathways [14,15].

Nowadays, the treatment of psoriasis mainly included topical treatment, systemic medications, and phototherapy [16,17]. However, these methods induced numerous side effects, such as relapse and adverse drug effects [17]. Since the pathological mechanism behind psoriasis was still not well understood, there was still a lack of safe, effective, and commonly accepted therapeutics [18]. Hence, it was urgent to find an agent with obvious therapeutic effect and low side effects in the treatment of psoriasis.

Numerous natural products with anti-inflammatory effects derived from medicinal plants, especially those used in traditional Chinese medicine, have been extensively used in clinical settings to prevent and treat many diseases with the advantages of minimal side effects and significant effectiveness [19,20]. The flowers of *Datura metel* L., namely *Flos daturae*, have been confirmed with obvious effects in the treatment of psoriasis because of their remarkable anti-inflammatory activity in our previous studies, and have been used in clinical settings [21,22,23,24,25,26]. Anolides and flavonoids were the main active ingredients of *Flos daturae* for psoriasis [22,23,24,25,26]. However, the precise mechanism by which the intragastric administration of *Flos daturae* exerted its anti-psoriatic effect remained unknown, which has restricted its widespread use.

Hence, this research was aimed at further proving and evaluating the anti-psoriasis effect of *Flos daturae* as well as exploring the roles of the TLR7/8–MyD88–NF-κB and NLRP3 pathways in its underlying mechanisms.

## 2. Results

### 2.1. The Effective Part of Datura metel *L.* (EPD) Treatment Ameliorated IMQ-Induced Psoriasis-Like Skin Lesions

**Figure 1:**
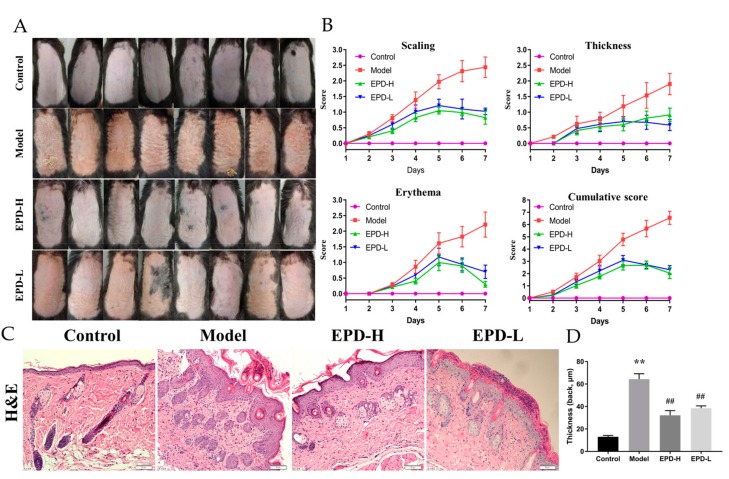
Effective part of *Datura metel* L. (EPD) improved the morphological and histological features of imiquimod (IMQ)-induced psoriasis dermatitis in mice. (**A**) Representative macroscopic views of the dorsal skin of C57BL/6 mice following continuous treatment for seven days. (**B**) Scaling, thickness, and erythema of the back skin was scored daily on a scale from 0 to 4. Additionally, the cumulative score (scaling plus thickness plus erythema) was depicted. (**C**) Histological evaluation of the back skin of IMQ-induced psoriasis-like mice (staining with hematoxylin and eosin, i.e., H&E; magnification 200×, scale bars indicate 50 μm). (**D**) Epidermal thickness of the dorsal skin on day 8. Data are represented as mean ± SD, n = 8. ** *P* < 0.01 vs. control group, ^##^ *P* < 0.01 vs. model group.

For investigating the protective effects of EPD on psoriasis mice induced by imiquimod (IMQ), EPD was orally administrated once daily 4 h after modeling for seven consecutive days, and the severity of lesions (thickening, scaling, erythema) on the back skin were scored on the basis of PASI. As shown in Figure 1A, the dorsal skin of the mice in the control group remained smooth and showed no morphological changes; however, compared to the control, typical symptoms of scaling, thickness, and erythema were observed after two to three days of modeling with IMQ, and presented a rough lesion resembling human plaque psoriasis. As expected, skin lesions in the EPD treatment groups were obviously alleviated in a dose-dependent manner, and displayed a significant improvement in scaling, thickening, and erythema as well as the reduced cumulative score compared to the model group (Figure 1B). The above results indicated that EPD significantly improved the clinical symptoms of the psoriasiform mice induced by IMQ.

The histopathological changes of EPD on the skin lesions induced by IMQ were evaluated used hematoxylin and eosin (H&E) staining (Figure 1C). The mice induced by IMQ showed pathological psoriatic lesions, including loss of the granular layer, epidermal hyperplasia, thickening of the acanthosis cell layer, and parakeratosis, as well as inflammatory cell infiltration. Compared to 13.01 ± 1.20 μm in the control (Figure 1D), the thickness of epidermis in the model was 64.36 ± 4.80 μm. Oral administration of a high dose and low dose of EPD could weaken most of these changes mentioned above; the thickness of epidermis were 32.18 ± 4.09 and 38.52 ± 2.06 μm, respectively.

### 2.2. EPD Inhibited the Aberrant Expression of the Proliferation and Differentiation Markers of Keratinocytes in Mice Dorsal Skin Induced by IMQ

**Figure 2:**
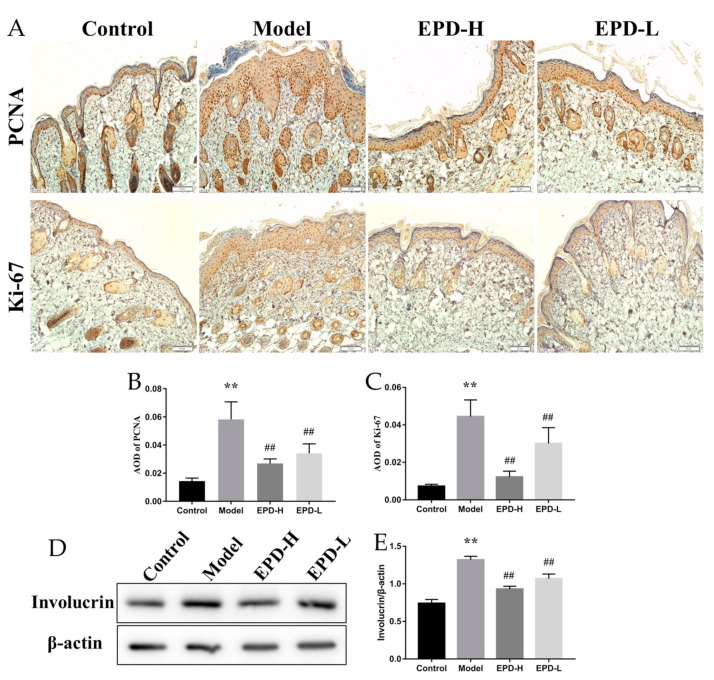
EPD inhibited the epidermal cell proliferation and differentiation of IMQ-induced psoriasis dermatitis mice models. (**A**) Expression of proliferating cell nuclear antigen (PCNA) and Ki-67 in each group were detected by immunohistochemistry (200×). (**B**,**C**) Average optical density (AOD) of PCNA and Ki-67 in each group. (**D**) The protein expressions of involucrin were detected using Western blot assay. (**E**) Quantification of protein levels of involucrin. Values are presented as the means ± SD (*n* = 3). ** *P* < 0.01 vs. control group, ^##^ *P* < 0.01 vs. model group.

Studies have revealed that proliferating cell nuclear antigen (PCNA) was an important indicator of cell mitosis and expressed in proliferative cells, especially basal cells, while Ki-67 was the hallmark protein involved in cell proliferation [2]. To investigate the relevance of the therapeutic effects of EPD on psoriasis and the inhibition of the hyperproliferation of keratinocyte, the expression of PCNA and Ki-67 in the lesional skin of mice were detected by immunohistochemistry. The quantification of positive immunostaining cells showed that abundant amounts of activated PCNA and Ki-67 were significantly observed in the model compared to the control group (*P* < 0.01); however, the expression of PCNA and Ki-67 in the skin lesions were reduced after treating with EPD, and these findings indicated that EPD reduced the IMQ-induced abnormal proliferation and differentiation of keratinocytes (Figure 2A–C).

Involucrin was a marker of epidermal differentiation and played a key role in the skin barrier function of the epidermis, which was mainly expressed in the cytoplasm [27,28]. Western blot analyses reveled that the expression of involucrin was significantly higher in the model compared to the control (*P* < 0.01). However, EPD treatment (both for group with a high dose of EPD, or EPD-H, and low dose of EPD, or EPD-L) remarkably decreased involucrin expression (*P* < 0.01, Figure 2D,E).

### 2.3. EPD Inhibited Inflammatory Cell Infiltration in IMQ-Induced Psoriasis-Like Mouse Mode

**Figure 3:**
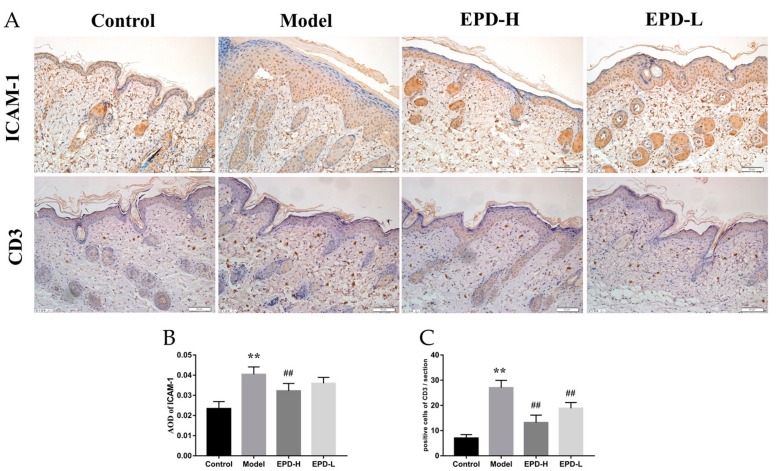
EPD inhibited inflammatory cell infiltration in IMQ-induced psoriasis-like mouse mode. (**A**) The expression of intercellular adhesion molecule 1 (ICAM-1) and CD3 in each group were detected by immunohistochemistry (200×). (**B**) AOD of PCNA in each group. (**C**) Quantification of CD3-positive cells in skin tissues. Values are presented as the means ± SD (*n* = 3). ** *P* < 0.01 vs. Control group, ^##^ *P* < 0.01 vs. Model group.

The infiltration of leukocytes into the skin could be regarded as one of characteristics of inflammatory immune responses in the psoriasis, and the increment of intercellular adhesion molecule 1 (ICAM-1) expression in the psoriatic lesions could improve the infiltration of leukocytes [29]. In our study, the expression of ICAM-1 was remarkably increased in the model group, and this trend was reversed through EPD treatment (*P* < 0.01, Figure 3A,B). Moreover, CD3+ T cells play an important role in the skin lesion in psoriasis [30]. Our immunohistochemical results revealed that CD3 expression in the skin of the model group mice was significantly higher than that of the control group. Importantly, a significant decrease in CD3 expression was observed in mice treated with EPD (*P* < 0.01, Figure 3A–C).

### 2.4. EPD Administration Downregulated the Expression of Inflammatory Cytokines

**Figure 4:**
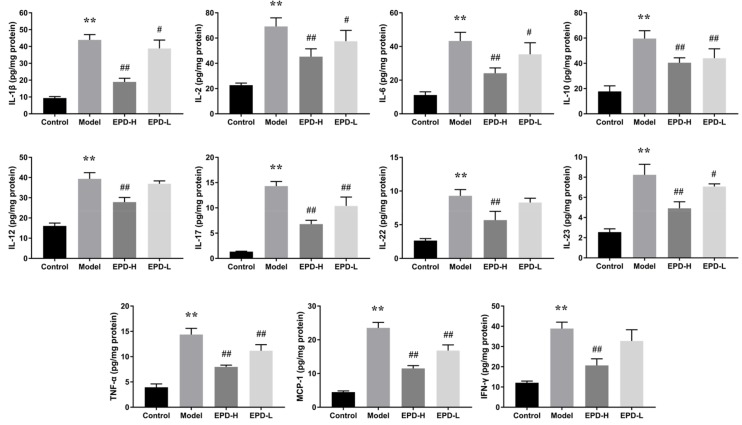
The concentrations of inflammatory cytokines in the dorsal skin of IMQ-induced psoriasis dermatitis in mice were measured with the corresponding ELISA kits. Vales are mean ± SD (*n* = 8 mice per group). ** *P* < 0.01 vs. control group, ^#^ *P* < 0.05, ^##^ *P* < 0.01 vs. model group.

The expressions of crucial inflammatory mediators (including IL-1β, IL-2, IL-6, IL-10, IL-17, IL-22, IL-23, IFN-γ, TNF-α) in skin tissues were measured by ELISA due to the import role of inflammatory cytokines in psoriasis. Our results showed that the levels of IL-1β, IL-2, IL-6, IL-10, IL-12, IL-17, IL-22, IL-23, IFN-γ, monocyte chemotactic protein 1 (MCP-1), and tumor necrosis factor-α (TNF-α) in the skin of mice were all significantly increased after IMQ treatment, whereas the expressions of these pro-inflammatory cytokines were dramatically reduced in a dose-dependent manner after oral administration with EPD (Figure 4). Our experimental data displayed that regulating inflammatory cytokines might contribute to the treatment effect of EPD for psoriasis.

### 2.5. EPD Suppressed the TLR7/8–MyD88–NF-κB Signaling Pathways and Accordingly Exerted the Therapeutic Effect for Psoriasis

**Figure 5:**
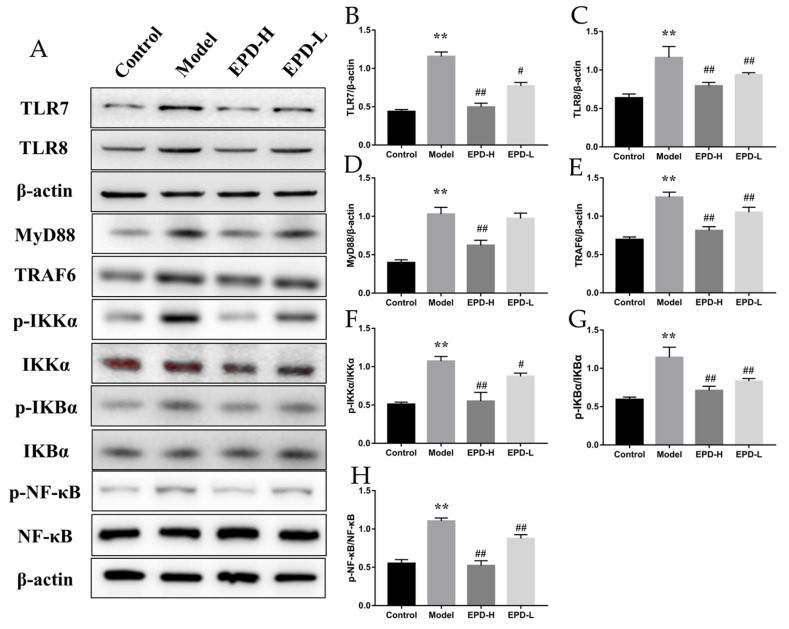
EPD suppressed the activation of the Toll-like receptor 7–myeloid differentiation primary response gene 88–nuclear factor-κB (TLR7/8–MyD88–NF-κB) signaling pathways. (**A**) The protein expressions of TLR7, TLR8, TRAF6, MyD88, p-IKKα, IKKα, p-IKBα, IKBα, p-NF-κB, and NF-κB were detected using Western blot assay. (**B**–**H**) The quantification of protein levels of TLR7, TLR8, TLAF6, MyD88, p-IKKα, IKKα, p-IKBα, IKBα, p-NF-κB, and NF-κB. Values are presented as the means ± SD of three independent experiments. ** *P* < 0.01 vs. control group, ^#^ *P* < 0.05, ^##^ *P* < 0.01 vs. model group.

Various studies presented evidence that the transduction pathway of the TLR7/8–MyD88–NF-κB signal was considered as an early event that was essential for inflammatory responses [28,31]. Thus, the protein expression levels of TLR7 and TLR8 in IMQ-induced psoriasis mice were detected. As shown in Figure 5, the TLR7 and TLR8 expression were increased by IMQ stimulation, which were not shown in the control group, and the increase was resisted by EPD treatment (*P* < 0.05). TLRs’ signaling involved the recruitment of the MyD88 adapter protein and eventually activated NF-κB; in our study, Western blot analysis was used to monitor the changes of downstream-signaling molecules related to TLR7/8 in skin tissues. The expressions of both TRAF6 and MyD88 were obviously elevated by IMQ treatment, as well as the phosphorylation level of IKKα, IKBα, and NF-κB in the model compared to control (*P* < 0.01), while the administration of EPD significantly reversed those (*P* < 0.01, Figure 5). The results indicated that the inhibition of the TLR7/8–MyD88–NF-κB pathway was relevant to the protective effects of EPD on IMQ-induced psoriasis.

### 2.6. EPD Inhibited the IMQ-Activated NLRP3 Inflammasome Signaling Pathway

**Figure 6:**
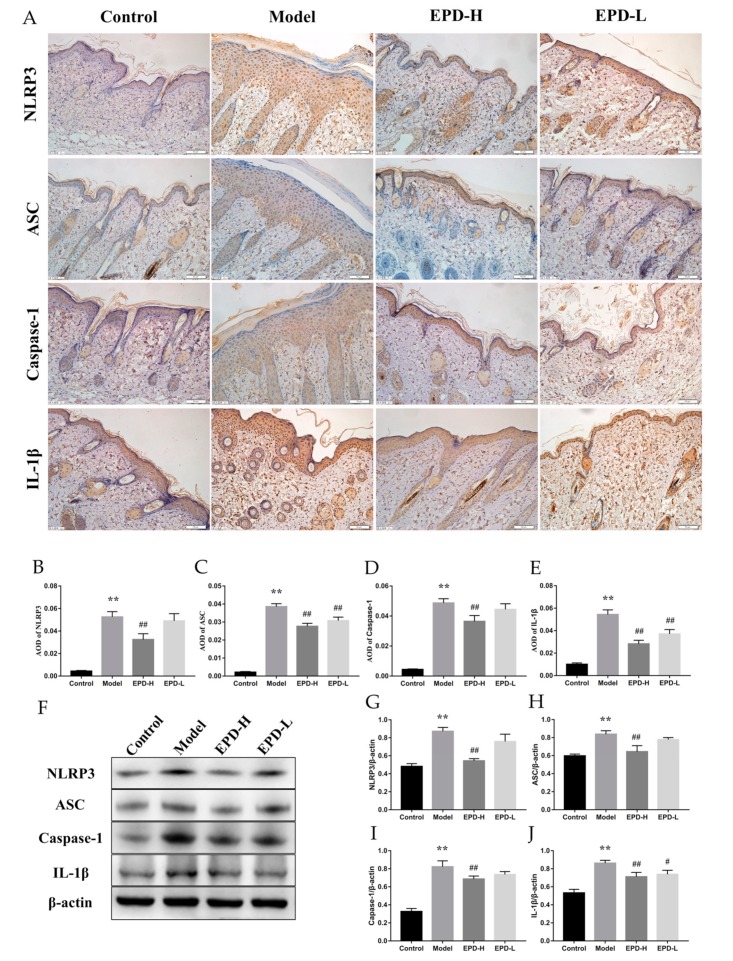
EPD inhibited the IMQ-activated nucleotide-binding oligomerization domain (Nod)-like receptor family pyrin domain-containing 3 (NLRP3) inflammasome signaling pathway. (**A**) The protein expressions of NLRP3, apoptosis-associated speck-like protein contained a caspase recruitment domain (ASC), cysteinyl aspartate specific proteinase 1 (caspase-1), and IL-1β were detected using immunohistochemistry (200×). (**B**–**E**) AOD of NLRP3, ASC, caspase-1 and IL-1β. Values are presented as the means ± SD (*n* = 6). (**F**) The protein expressions of NLRP3, ASC, caspase-1, and IL-1β were detected using Western blot assay. (**G**–**J**) Quantification of protein levels of NLRP3, ASC, caspase-1, and IL-1β. Values are presented as the mean ± SD of three independent experiments. ** *P* < 0.01 vs. control group, ^#^ *P* < 0.05, ^##^ *P* < 0.01 vs. model group.

The expression of inflammatory mediators was closely associated with the activation of the NLRP3 inflammasome [32]. Here, the proteins’ expression of the NLRP3 inflammasome pathway was determined by immunohistochemical and Western blot analysis. The immunohistochemical results showed that the NLRP3 inflammasome signals were observed to be significantly increased in the psoriasis mice; furthermore, the expressions of NLRP3, ASC, caspase-1, and IL-1β were upregulated, which were all inhibited through EPD treatment (Figure 6A–E). Similar results were also found using Western blotting (Figure 6F–J).

## 3. Discussion

Psoriasis is a skin disease that has been characterized as common, chronic, and recurring. The mechanisms that participate in the genesis of psoriasis are complex, and previous research studies demonstrated that the typical form of psoriasis exaggerated the infiltration of specific immune and inflammatory cells in the epidermis and dermis. These effects in turn sustained the inflammatory extent of the skin layer, and subsequently functionally increased the proliferation and aberrant differentiation of keratinocytes and the destruction of the skin [33,34]. Previously, we found that *Datura metel* L. had an obvious effect on psoriasis, and withanolides and flavonoids were the main constituents of the EPD [22,23,24]. A growing body of research also proved that withanolides and flavonoids exhibited excellent anti-inflammatory activity [35].

IMQ was both a potent immune activator and Toll-like receptor agonist. A high-dose topical application of IMQ repeatedly on mouse ear or dorsal skin could induce erythema, scaling, keratinocyte proliferation with acanthosis, and also increase the expressions of immune cells and their related cytokines; all of these features are consistent with psoriatic conditions [36,37]. Therefore, the IMQ-induced mice model was widely applied in the research studies on psoriasis [38]. Additionally, Swindell WR’s research demonstrated that the IMQ-induced dermatitis was strain-dependent, and C57BL/6 mice were more suitable for the psoriasis modeling and pathogenesis research because of their better genetic background than other strains [39]. In our study, the symptom of psoriasis-like lesions in animal C57BL/6 mice, which were induced by the topical administration of IMQ, was similar to that found in the literature [40,41]. The administration of EPD could lower scores of PASI and significantly attenuate the thickened epidermis in a psoriasis-like mice model. In addition, histological analyses showed that the mice treated with EPD exhibited smoother epidermis, less epidermal thickening, and parakeratosis. These findings suggested that EPD had remarkable treatment effects on a psoriasis-like mice model induced by IMQ. In the development of psoriasis, activated keratinocytes were considered to play a crucial role [41,42]. The results suggested that EPD obviously inhibited the expression of PCNA, Ki-67, and involucrin, which were the important markers of the proliferation or differentiation of keratinocytes [43]. Several studies indicated that the excessive proliferation of keratinocytes could lead to the activation of cytokine production, which was closely related to the psoriatic pathogenesis in the inflammatory regulation, and the levels of these cytokines were positively correlated with the severity of disease [44,45,46]. More importantly, the activated keratinocytes could produce large amounts of inflammatory cytokines, which induced proliferation and survival and in turn elevated keratinocyte proliferation and epidermal thickening, and then formed a positive loop to perpetuate the psoriatic lesions [36]. The data in our study results showed that the expression of inflammatory factors was markedly inhibited after the treatment of EPD in psoriasiform mice induced by IMQ.

Generally, imiquimod was an agonist for TLR7/8, and TLR7/8 signaling was essential for innate immune response inflammatory signaling pathways, and considered to be involved in the pathology of imiquimod-induced psoriasis-like dermatitis [41]. Once activated by their ligands, TLR7/8 played a regulatory role in the inflammation by interacting with MyD88 and the Toll receptor-associated activator of interferon, which were two distinct adaptor proteins. Activated TLR7/8 promoted the expression of MyD88 and influenced the expression of its downstream protein TRAF6, as well as increased the level of inflammatory factors with the upregulation of MyD88 and TRAF6. In turn, these inflammatory factors played a vital role in the TLR-mediated MyD88-dependent pathway [47]. Moreover, activated TRAF6 degraded the inhibitor of NF-κB kinases (IKKs), led to phosphorylation, and also degraded the IκB-α, which was the inhibitory protein of NF-κB. Then, the NF-κB translocated into the nucleus in order to trigger NF-κB signaling pathways after separating from IκB-α, which had been reported to be involved in the pathological process of IMQ-induced psoriasis lesions [48,49]. Our present study showed that the protein expression levels of TLR7, TLR8, MyD88, and p-NF-κB in the skin of mice induced by IMQ were upregulated significantly; consequently, TLR7/8–MyD88–NF-κB signaling was considered to play a critical role in IMQ-induced psoriasis. It was worth noting that all of these changes were obviously reversed by EPD treatment, which provided evidence that TLR7/8–MyD88–NF-κB signaling was involved in the therapeutic action of EPD on psoriasis.

The NLRP3 inflammasome was an important intracellular multiprotein inflammatory pathway and governed the productions of pro-inflammatory cytokines [14,50]. The NLRP3 inflammasome consisted of three sections, including NLRP3, ASC, and caspase-1. Ample evidence had shown that NLRP3 was particularly activated in psoriasis, and it was expressed both in the inflammatory cellular infiltrate in the dermis and in the epithelial cells of the psoriatic epidermis. The activation of NLRP3 triggered a cascade process, including ASC and pro-caspase-1 recruitment, caspase-1 activation, and pro-IL-1β being cleavaged into its mature form. Here, the activated NLRP3 inflammasome as an upstream trigger could govern the pathogenesis of tissular inflammatory injuries in psoriasis [51]. Thus, the targeted inhibition of the NLRP3 pathway, and subsequent reducing inflammatory injury might be an effective method of psoriasis treatment. The results from our current experiments suggested that the oral administration of EPD effectively downregulated the expressions of IL-1β and ASC caspase-1 as well as NLRP3.

## 4. Materials and Methods

### 4.1. The Effective Part of Datura metel *L.* (EPD) Preparation

The EPD was prepared according to our previous study [52]. Briefly, dried *Flos daturae* were refluxed with 70% alcohol (1:30, *w*/*v*, 120 min × 3). Then, the extract was acidified with 0.1% hydrochloric acid. After filtration, the filtrate was subjected to 732 cation exchange resin; then, the effluent was chromatographed on AB-8 macroporous resin and eluted by H_2_O, 50% ethanol, and 95% ethanol, respectively. Then, 50% ethanol eluate was collected and vacuum evaporated to obtain dried powder, which was regarded as the EPD for psoriasis. Kaempferol-3-O-β-d-glucopyranosyl-(1→2)-β-d-galacto-pyranosyl-7-O-α-l-rhamnopyranoside and daturataturin A were used as control standards; the contents of the total flavonoids and total withanolides of EPD were determined by ultraviolet-visible spectrophotometry, with contents of 55.25% and 23.26%, respectively [53,54]. EPD was stored at 4 °C before use and dissolved homogeneously by using saline when administrated.

### 4.2. Experimental Animal

Male C57BL/6 mice (No. SCXK(JING) 2016-0006; Beijing Vital River Laboratory Animal Technology Co., Ltd., China) 7 weeks to 8 weeks old, weighing between 20–24 g, were used in the current study. In addition, all the mice were allowed to acclimatize to drink and eat at will and housed under controlled conditions (maintained at 23 ± 2 °C, 40% to 50% relative humidity) for 7 days before the start of the experiment.

### 4.3. Establishment of Psoriasis-Like Dermatitis Mice Model and the Administration with EPD

**Figure 7:**
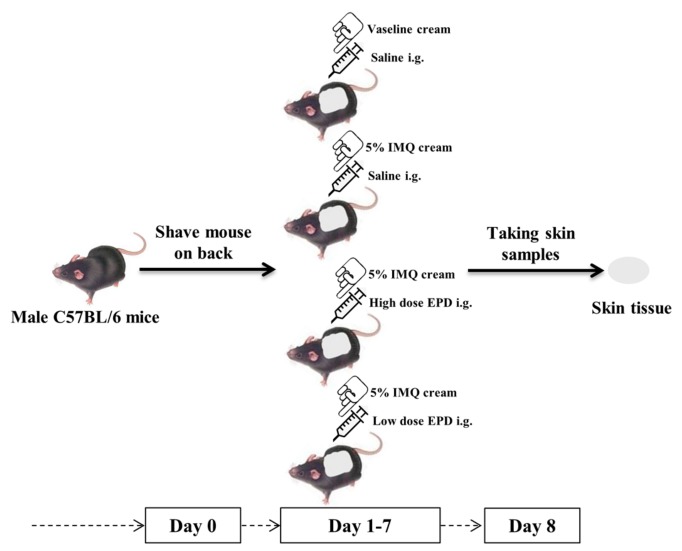
Schematic representation of the animal experiment protocol.

The experimental process was strictly approved by the Animal Ethics Committee of Heilongjiang University of Traditional Chinese Medicine (Heilongjiang, China) on 14 September 2017 (number 2017091403). Healthy male C57BL/6 mice (*n* = 32) were assigned into four groups after shaved to dorsal skin randomly: control, model, high dose of EPD (EPD-H), and low dose of EPD (EPD-L). According to the previous study, 62.5 mg of commercially available IMQ cream (Sichuan Mingxin Pharmaceutical Co., Ltd., Chengdu, China) was applied topically once per day to a 2 cm × 3 cm shaved area dorsal skin of mice in the model, EPD-H, and EPD-L groups for 7 days in a row to induce a psoriasis-like mice model [39]. Mice in the control were treated similarly with petroleum jelly to the same skin sites according to the same schedule. The treatment and experimental design are shown in Figure 7. The mice in the EPD-H and EPD-L groups received an intragastric administration of EPD 104 mg/kg/day and 52 mg/kg/day after 4 h of each modeling [23]. Furthermore, the mice in the control and model received oral administration with the same volume of saline.

### 4.4. Psoriasis Area and Severity Index (PASI) Assessment

Based on PASI, the severity of the skin inflammation was evaluated once daily, including the measurements for skin erythema, scaling, thickening and scored separately using a five-point scale from 0 to 4, namely: 0, none; 1, slight; 2, moderate; 3, marked; and 4, very marked [36].

### 4.5. Histological Analysis

After 24 h of final modeling, the dorsal skin tissues were harvested from mice and excised on ice. Then, part of the excised skin samples were washed with saline and wiped. Then, after liquid nitrogen freezing, the skin samples were stored under −80 °C and used for the further experiments. The other part of the skin samples was fixed in 10% neutral formalin, embedded in paraffin, and sectioned into 5-μm thick sections and stained with hematoxylin and eosin (H&E) under a light microscope for pathological observation (OLYMPUS BX60, 200× magnification). The average optical density (AOD) of related indicators and acanthosis (epidermal thickness) of skin were measured by image analysis software (OLYMPUS DP72).

### 4.6. Immunohistochemistry

The expression of proliferating cell nuclear antigen (PCNA), nuclear-associated antigen Ki-67 (Ki-67), intercellular adhesion molecule 1 (ICAM-1), ASC, caspase-1, and IL-1β in the lesional skin of mice were detected by immunohistochemistry. In brief, the paraffin-embedded sections were dewaxed and rehydrated for antigen retrieval. Then, 3% hydrogen peroxide and phosphate-buffered saline were used to block endogenous peroxidase and nonspecific sites (reactive sites). Then, paraffin sections were incubated overnight with rabbit polyclonal anti-PCNA (1:500, bs-2007R, Bioss), rabbit polyclonal anti-NLRP3 (1:400, bs-10021R, Bioss), rabbit polyclonal anti-Ki-67 (1:500, bs-23103R, Bioss), rabbit polyclonal anti-ICAM-1 (1:300, bs-0608R, Bioss), rabbit monoclonal anti-CD3 (1:50, ab16669, Abcam), rabbit polyclonal anti-ASC (1:400, bs-6741R, Bioss), rabbit polyclonal anti-caspase-1 (1:50, ab1872, Abcam), rabbit polyclonal anti-IL-1β (1:400, bs-0812R, Bioss), and stained with a rabbit two-step detection kit (PV-9001, Beijing Zhongshan Jinqiao biological company) according to the manufacturer’s instructions. The staining was assessed according to the method described above.

### 4.7. Detection of Inflammatory Cytokines in Skin Tissues Lysate

Skin samples were ground with liquid nitrogen, and the Tissue Protein Extraction Reagent (78510, Thermo Fisher Scientific, MA, USA) was then used to extract the skin protein. The extracted proteins were centrifuged for 20 min (12,000 rpm). Subsequently, the supernatant was collected, and the concentrations of protein were measured using the bicinchoninic acid (BCA) protein assay kit (23227, Thermo Fisher Scientific). Commercially available enzyme-linked immunosorbent assay (ELISA) kits were used to determine the expression of cytokines, including IL-1β, IL-2, IL-6, IL-10, IL-12, IL-17, IL-22, IL-23, tumor necrosis factor-α (TNF-α), and monocyte chemotactic protein 1 (MCP-1) as well as interferon-γ (IFN-γ) (CUSABIO BIOTECH, Ltd. Wuhan, China); all the procedures were strictly according to the instructions of manufacturer. 

### 4.8. Western Blot Analysis

The total protein of skin samples were obtained with radioimmunoprecipitation assay (RIPA) lysis buffer containing phosphatase inhibitors and a protease inhibitor cocktail at 4 °C (1 h). After centrifuging at 4 °C (10 min, 12,000 rpm), the supernatant was collected, and the protein concentration was determined in the same way as mentioned above. Samples were mixed with 4× electrophoresis sample buffer solution with bromophenol blue and boiled for 10 min at 95 °C. Then, equal amounts protein samples were separated by 10% sodium dodecyl sulfate-polyacrylamide gel electrophoresis (SDS-PAGE) gel and then electrotransferred to a polyvinylidene fluoride (PVDF) membrane. After being blocked with 5% (*w*/*v*) bovine serum albumin (BSA) in Tris-buffered saline Tween-20 (TBST) at room temperature for 1 h, the membrane was incubated with primary antibodies, including rabbit monoclonal anti-TLR7 (1:1000, ab124928, Abcam), rabbit polyclonal anti-TLR8 (1:1500, ab180610, Abcam), rabbit polyclonal anti-Involucrin (1:800, bs-23062R, Bioss), rabbit monoclonal anti-TRAF6 (1:1000, ab33915, Abcam), rabbit polyclonal anti-MyD88 (1:1000, ab2064, Abcam), rabbit polyclonal anti-phospho-IKKα (1:500, ab38515, Abcam), rabbit polyclonal anti-IKBα (1:500, bs-1287R, Bioss), rabbit monoclonal anti-IKKα (1:5000, ab32041, Abcam), rabbit polyclonal anti-phospho-IKBα (1:500, AP0614, Abclonal), rabbit monoclonal anti-phospho-NF-κB (1:1000, 3033, CST), rabbit monoclonal anti-NF-κB (1:1000, 8242, CST), rabbit polyclonal anti-NLRP3 (1:300, bs-10021R, Bioss), rabbit polyclonal anti-ASC (1:500, bs-6741R, Bioss), rabbit polyclonal anti-caspase-1 (1:500, ab1872, Abcam), rabbit polyclonal anti-IL-1β (1:500, bs-0812R, Bioss), and rabbit polyclonal anti-β-Actin (1:3000, AC026, abclonal) at 4 °C overnight and then washed three times using TBST, and incubated with HPR-conjugated secondary antibody for 1 h (1:3000, LK2001, Affinity). Finally, the membranes were triple-washed with TBST; then, an enhanced chemiluminescence (ECL) kit (KF001, Affinity) was used to detect the signals, which were visualized by the Odyssey Infrared Imaging System (LI-COR, Inc., Lincoln, NE, USA) and analyzed by Image J software.

### 4.9. Statistical Analysis

The data were all expressed as mean ± SD. All the standard statistical analysis was performed using GraphPad Prism Version 7.0 (GraphPad Software, La Jolla, CA, USA) software, including one-way Analysis of Variance (ANOVA) followed by multiple comparisons between groups using Tukey’s post-hoc test. Statistically significant differences were identified as either *P* less than 0.05 or *P* less than 0.01.

## 5. Conclusions

**Figure 8:**
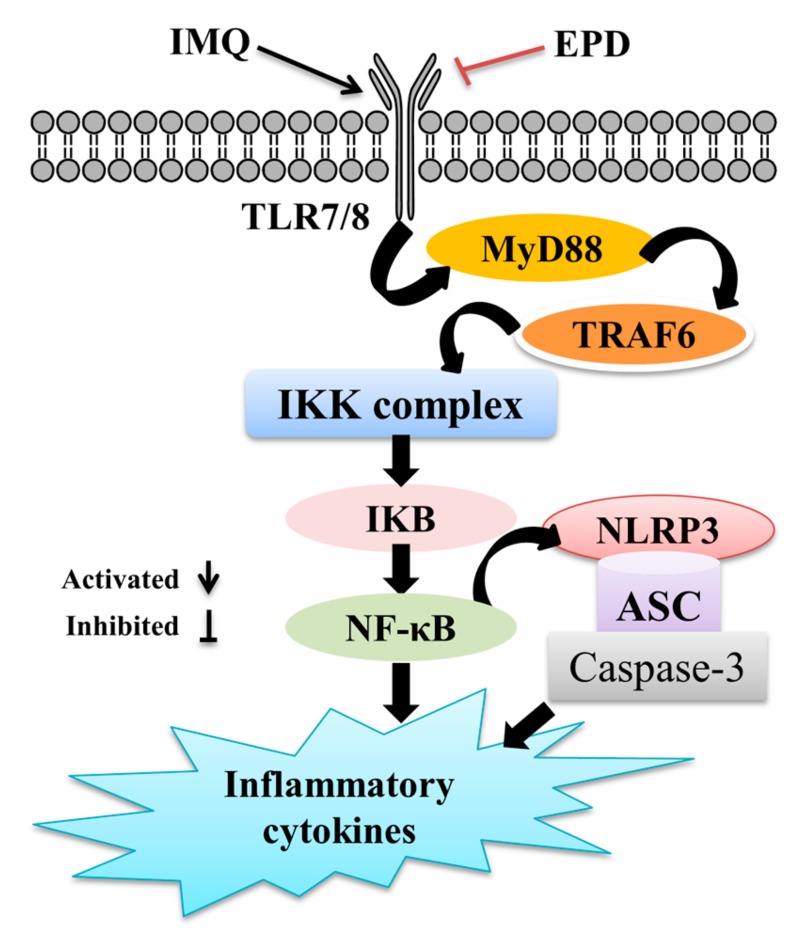
Schematic diagram of the mechanism by which EPD attenuates IMQ-induced psoriasis by inhibiting the inflammation responses via TLR7/8–MyD88–NF-κB–NLRP3 inflammasome signaling pathways.

The mouse model of psoriasis-like dermatitis induced by IMQ was first used for evaluating the therapeutic effect of EPD for psoriasis, and exploring the mechanism of the inflammation-signaling pathway. Our study indicated that EPD could alleviate psoriasis in the IMQ-induced mice model and that the potential mechanisms that were involved were, at least in part, due to inhibiting the inflammation responses via the TLR7/8–MyD88–NF-κb–NLRP3 inflammasome signaling pathway (Figure 8). Our findings provided the more scientific information in vivo about a clinical value of *Datura metel* L. for psoriasis.

## References

[1] Terhorst D., Chelbi R., Wohn C., Malosse C., Tamoutounour S., Jorquera A., Bajenoff M., Dalod M., Malissen B., Henri S. (2015). Dynamics and Transcriptomics of Skin Dendritic Cells and Macrophages in an Imiquimod-Induced, Biphasic Mouse Model of Psoriasis. J. Immunol..

[2] Meng Y., Wang M., Xie X., Di T., Zhao J., Lin Y., Xu X., Li N., Zhai Y., Wang Y. (2017). Paeonol ameliorates imiquimod-induced psoriasis-like skin lesions in BALB/c mice by inhibiting the maturation and activation of dendritic cells. Int. J. Mol. Med..

[3] Ryan C., Kirby B. (2015). Psoriasis is a systemic disease with multiple cardiovascular and metabolic comorbidities. Dermatol. Clin..

[4] Gutmark-Little I., Shah K.N. (2015). Obesity and the metabolic syndrome in pediatric psoriasis. Clin. Dermatol..

[5] Chularojanamontri L., Wongpraparut C., Silpa-Archa N., Chaweekulrat P. (2016). Metabolic syndrome and psoriasis severity in South-East Asian patients: An investigation of potential association using current and chronological assessments. J. Dermatol..

[6] Tsai T.F., Wang T.S., Hung S.T., Tsai P.I., Schenkel B., Zhang M., Tang C.H. (2011). Epidemiology and comorbidities of psoriasis patients in a national database in Taiwan. J. Dermatol. Sci..

[7] Davidovici B.B., Sattar N., Prinz J., Puig L., Emery P., Barker J.N., van de Kerkhof P., Stahle M., Nestle F.O., Girolomoni G. (2010). Psoriasis and systemic inflammatory diseases: Potential mechanistic links between skin disease and co-morbid conditions. J. Invest. Dermatol..

[8] Obradors M., Blanch C., Comellas M., Figueras M., Lizan L. (2016). Health-related quality of life in patients with psoriasis: A systematic review of the European literature. Qual. Life Res..

[9] De Korte J., Sprangers M.A., Mombers F.M., Bos J.D. (2004). Quality of life in patients with psoriasis: A systematic literature review. J. Investig. Dermatol. Symp. Proc..

[10] Buchanan M.M., Hutchinson M., Watkins L.R., Yin H. (2010). Toll-like receptor 4 in CNS pathologies. J. Neurochem..

[11] Kawakita F., Fujimoto M., Liu L., Nakano F., Nakatsuka Y., Suzuki H. (2017). Effects of Toll-Like Receptor 4 Antagonists Against Cerebral Vasospasm After Experimental Subarachnoid Hemorrhage in Mice. Mol. Neurobiol..

[12] Avlas O., Fallach R., Shainberg A., Porat E., Hochhauser E. (2011). Toll-like receptor 4 stimulation initiates an inflammatory response that decreases cardiomyocyte contractility. Antioxid. Redox Signal..

[13] Zhang Y., Li X., Grailer J.J., Wang N., Wang M., Yao J., Zhong R., Gao G.F., Ward P.A., Tan D.X. (2016). Melatonin alleviates acute lung injury through inhibiting the NLRP3 inflammasome. J. Pineal Res..

[14] Carlström M., Ekman A.K., Petersson S., Söderkvist P., Enerbäck C. (2012). Genetic support for the role of the NLRP3 inflammasome in psoriasis susceptibility. Exp. Dermatol..

[15] Yu P., Hao S., Zheng H., Zhao X., Li Y. (2018). Association of NLRP1 and NLRP3 Polymorphisms with Psoriasis Vulgaris Risk in the Chinese Han Population. Biomed. Res. Int..

[16] Sun L., Liu Z., Wang L., Cun D., Tong H.H.Y., Yan R., Chen X., Wang R., Zheng Y. (2017). Enhanced topical penetration, system exposure and anti-psoriasis activity of two particle-sized, curcumin-loaded PLGA nanoparticles in hydrogel. J. Control. Release..

[17] Okasha E.F., Bayomy N.A., Abdelaziz E.Z. (2018). Effect of topical application of black seed oil on imiquimod-induced psoriasis-like lesions in the thin skin of adult male albino rats. Anat. Rec. (Hoboken).

[18] Jeon Y.J., Sah S.K., Yang H.S., Lee J.H., Shin J., Kim T.Y. (2017). Rhododendrin inhibits toll-like receptor-7-mediated psoriasis-like skin inflammation in mice. Exp. Mol. Med..

[19] Li N., Zhao W., Xing J., Liu J., Zhang G., Zhang Y., Li Y., Liu W., Shi F., Bai Y. (2017). Chinese herbal Pulian ointment in treating psoriasis vulgaris of blood-heat syndrome: A multi-center, double-blind, randomized, placebo-controlled trial. BMC Complement. Altern. Med..

[20] Meng S., Lin Z., Wang Y., Wang Z., Li P., Zheng Y. (2018). Psoriasis therapy by Chinese medicine and modern agents. Chin. Med..

[21] Yang B.Y., Xia Y.G., Wang Q.H., Dou D.Q., Kuang H.X. (2010). Baimantuoluosides D-G, four new withanolide glucosides from the flower of Datura metel L.. Arch. Pharm. Res..

[22] Yang B.Y., Guo R., Li T., Liu Y., Wang C.F., Shu Z.P., Wang Z.B., Zhang J., Xia Y.G., Jiang H. (2014). Five withanolides from the leaves of Datura metel L. and their inhibitory effects on nitric oxide production. Molecules.

[23] Wang Q.H., Xiao H.B., Yang B.Y., Yao F.Y., Kuang H.X. (2008). Studies on pharmacological actions of the effective parts for psoriasis in Flos Daturae (I)-The Anti-inflammatory, Anti-titillation and Anti-anaphylaxis Actions of Flos daturae. Chin. J. Exp Trad Med. Formulae.

[24] Wang Q.H., Xiao H.B., Yang B.Y., Yao F.Y., Kuang H.X. (2008). Studies on Pharmacological actions of the active parts in Flos Daturae for psoriasis (II)-on Immune Funct ion, Epithel ial Cell Mitos is and Skin Keratosis. Chin. J. Exp. Trad. Med. Formulae.

[25] Kuang H.X., Yang B.Y., Xia Y.G., Wang Q.H. (2011). Two new withanolide lactones from flos daturae. Molecules.

[26] Yang B.Y., Xia Y.G., Wang Y.Y., Wang Q.H., Kuang H.X. (2013). Two novel norwithasteroids with unusual six- and seven-membered ether rings in side chain from flos daturae. Evid. Based Complement. Alternat. Med..

[27] Ferreira F.R., Di Chiacchio N.G., Alvarenga M.L., Mandelbaum S.H. (2013). Involucrin in the differential diagnosis between linear psoriasis and inflammatory linear verrucous epidermal nevus: A report of one case. An. Bras. Dermatol..

[28] Son E.D., Kim H.J., Kim K.H., Bin B.H., Bae I.H., Lim K.M., Yu S.J., Cho E.G., Lee T.R. (2016). S100A7 (psoriasin) inhibits human epidermal differentiation by enhanced IL-6 secretion through IκB/NF-κB signalling. Exp. Dermatol..

[29] Kim H., Youn G.S., An S.Y., Kwon H.Y., Choi S.Y., Park J. (2016). 2,3-Dimethoxy-2’-hydroxychalcone ameliorates TNF-α-induced ICAM-1 expression and subsequent monocyte adhesiveness via NF-kappaB inhibition and HO-1 induction in HaCaT cells. BMB Rep..

[30] Stockenhuber K., Hegazy A.N., West N.R., Ilott N.E., Stockenhuber A., Bullers S.J., Thornton E.E., Arnold I.C., Tucci A., Waldmann H. (2018). Foxp3+ T reg cells control psoriasiform inflammation by restraining an IFN-I-driven CD8+ T cell response. J. Exp. Med..

[31] Peng L., Li Q., Wang H., Wu J., Li C., Liu Y., Liu J., Xia L., Xia Y. (2018). Fn14 deficiency ameliorates psoriasis-like skin disease in a murine model. Cell Death Dis..

[32] Yu N., Liu S., Yi X., Zhang S., Ding Y. (2015). Serum amyloid A induces interleukin-1β secretion from keratinocytes via the NACHT, LRR and PYD domains-containing protein 3 inflammasome. Clin. Exp. Immunol..

[33] Bech R., Jalilian B., Agger R., Iversen L., Erlandsen M., Otkjaer K., Johansen C., Paludan S.R., Rosenberg C.A., Kragballe K. (2016). Interleukin 20 regulates dendritic cell migration and expression of co-stimulatory molecules. Mol. Cell. Ther..

[34] Hung C.H., Wang C.N., Cheng H.H., Liao J.W., Chen Y.T., Chao Y.W., Jiang J.L., Lee C.C. (2018). Baicalin Ameliorates Imiquimod-Induced Psoriasis-Like Inflammation in Mice. Planta Med..

[35] Sang-Ngern M., Youn U.J., Park E.J., Kondratyuk T.P., Simmons C.J., Wall M.M., Ruf M., Lorch S.E., Leong E., Pezzuto J.M. (2016). Withanolides derived from Physalis peruviana (Poha) with potential anti-inflammatory activity. Bioorg. Med. Chem. Lett..

[36] Chen H., Lu C., Liu H., Wang M., Zhao H., Yan Y., Han L. (2017). Quercetin ameliorates imiquimod-induced psoriasis-like skin inflammation in mice via the NF-kappaB pathway. Int. Immunopharmacol..

[37] Van der Fits L., Mourits S., Voerman J.S., Kant M., Boon L., Laman J.D., Cornelissen F., Mus A.M., Florencia E., Prens E.P. (2009). Imiquimod-induced psoriasis-like skin inflammation in mice is mediated via the IL-23/IL-17 axis. J. Immunol..

[38] Lin Y.K., Yang S.H., Chen C.C., Kao H.C., Fang J.Y. (2015). Using Imiquimod-Induced Psoriasis-Like Skin as a Model to Measure the Skin Penetration of Anti-Psoriatic Drugs. PLoS ONE.

[39] Swindell W.R., Michaels K.A., Sutter A.J., Diaconu D., Fritz Y., Xing X., Sarkar M.K., Liang Y., Tsoi A., Gudjonsson J.E. (2017). Imiquimod has strain-dependent effects in mice and does not uniquely model human psoriasis. Genome Med..

[40] Li X., Xie X., Zhang L., Meng Y., Li N., Wang M., Zhai C., Liu Z., Di T., Zhang L. (2019). Hesperidin inhibits keratinocyte proliferation and imiquimod-induced psoriasis-like dermatitis via the IRS-1/ERK1/2 pathway. Life Sci..

[41] Li Y., Zhang G., Chen M., Tong M., Zhao M., Tang F., Xiao R., Wen H. (2019). Rutaecarpine inhibited imiquimod-induced psoriasis-like dermatitis via inhibiting the NF-κB and TLR7 pathways in mice. Biomed Pharmacother..

[42] Wang C., Zong J., Li Y., Wang X., Du W., Li L. (2019). MiR-744-3p regulates keratinocyte proliferation and differentiation via targeting KLLN in psoriasis. Exp. Dermatol..

[43] Johnson-Huang L.M., Lowes M.A., Krueger J.G. (2012). Putting together the psoriasis puzzle: An update on developing targeted therapies. Dis. Model. Mech..

[44] Alalaiwe A., Hung C.F., Leu Y.L., Tahara K., Chen H.H., Hu K.Y., Fang J.Y. (2018). The active compounds derived from Psoralea corylifolia for photochemotherapy against psoriasis-like lesions: The relationship between structure and percutaneous absorption. Eur. J. Pharm. Sci..

[45] Eyerich K., Dimartino V., Cavani A. (2017). IL-17 and IL-22 in immunity: Driving protection and pathology. Eur. J. Immunol..

[46] Kitahata K., Matsuo K., Hara Y., Naganuma T., Oiso N., Kawada A., Nakayama T. (2018). Ascorbic acid derivative DDH-1 ameliorates psoriasis-like skin lesions in mice by suppressing inflammatory cytokine expression. J. Pharmacol. Sci..

[47] Wang M.X., Zhao J.X., Meng Y.J., Di T.T., Xu X.L., Xie X.J., Lin Y., Zhang L., Wang N., Li P. (2018). Acetyl-11-keto-β-boswellic acid inhibits the secretion of cytokines by dendritic cells via the TLR7/8 pathway in an imiquimod-induced psoriasis mouse model and in vitro. Life Sci..

[48] Xu J., Duan X., Hu F., Poorun D., Liu X., Wang X., Zhang S., Gan L., He M., Zhu K. (2018). Resolvin D1 attenuates imiquimod-induced mice psoriasiform dermatitis through MAPKs and NF-κB pathways. J. Dermatol. Sci..

[49] Dou R., Liu Z., Yuan X., Xiangfei D., Bai R., Bi Z., Yang P., Yang Y., Dong Y., Su W. (2017). PAMs ameliorates the imiquimod-induced psoriasis-like skin disease in mice by inhibition of translocation of NF-κB and production of inflammatory cytokines. PLoS ONE.

[50] Irrera N., Vaccaro M., Bitto A., Pallio G., Pizzino G., Lentini M., Arcoraci V., Minutoli L., Scuruchi M., Cutroneo G. (2017). BAY 11-7082 inhibits the NF-κB and NLRP3 inflammasome pathways and protects against IMQ-induced psoriasis. Clin. Sci. (Lond.).

[51] Han W., Ma Q., Liu Y., Wu W., Tu Y., Huang L., Long Y., Wang W., Yee H., Wan Z. (2018). Huangkui capsule alleviates renal tubular epithelial-mesenchymal transition in diabetic nephropathy via inhibiting NLRP3 inflammasome activation and TLR4/NF-κB signaling. Phytomedicine.

[52] Yang L.R., Meng X., Kuang H.X. (2018). Comparisons of the pharmacokinetic and tissue distribution profiles of withanolide B after intragastric administration of the effective part of Datura metel L. in normal and psoriasis guinea pigs. J. Chromatogr. B.

[53] Tang L., Wand Q.H., Yang B.Y., Xiao H.B., Sun Y.P., Kuang H.X. (2006). Protective ef fects of active fraction and const ituents from Flos Daturae on Chinese hamster ovary cells injuried by dimethyl sulfoxide. Chin. Trad. Herbal Drugs.

[54] Guo R., Yang B.Y., Li T., Wang Q.H., Kuang H.X. (2015). Optimization of Extraction Technology for the Leaves of Datura Metel, L. by Central Composition Design-Response Surface Methodology. Inform. Trad. Chin. Med..

